# Exploring middle-aged adults’ satisfaction with the Wasfaty electronic prescription system: a cross-sectional study in Tabuk, Saudi Arabia

**DOI:** 10.7717/peerj.21011

**Published:** 2026-03-23

**Authors:** Kousalya Prabahar

**Affiliations:** Department of Pharmacy Practice, Faculty of Pharmacy, University of Tabuk, Tabuk, Saudi Arabia

**Keywords:** Wasfaty system, Middle-aged adults, Perception, Satisfaction

## Abstract

**Background:**

Patient perspectives and satisfaction serve as key indicators of pharmacy service quality. The Wasfaty electronic prescription system is a flagship service within Saudi Arabia’s ongoing Vision 2030 healthcare reforms, designed to enhance efficiency, accuracy, and convenience for patients and providers. This study aims to assess middle-aged adults’ perceptions and satisfaction with the Wasfaty system in Tabuk, Saudi Arabia. The secondary aim was to explore the association between satisfaction and various demographic factors.

**Methods:**

From February to April 2025, a cross-sectional survey was conducted among middle-aged adults attending selected community pharmacies in Tabuk, Saudi Arabia, using a convenience sampling approach. The self-administered questionnaire included sections on demographics, health status, availability of Wasfaty services and medications, pharmacists’ counseling practices, and overall satisfaction with the Wasfaty electronic prescription system. Data were analyzed with SPSS v27.0, using descriptive statistics (frequencies, percentages) to summarize participant characteristics and response patterns. Logistic regression models yielded crude odds ratios (CORs) and adjusted odds ratios (AORs), each with 95% confidence intervals. Statistical significance was defined as *p* < 0.05.

**Results:**

Of 400 respondents (response rate = 97%), 54.3% were male and 61% aged 40–49, with 63.3% reporting chronic conditions. Eighty percent expressed satisfaction with the Wasfaty system. Satisfaction was significantly linked to service proximity, pharmacists’ guidance on proper medication use, and system efficiency. Men had significantly higher odds of positive views regarding pharmacists’ counseling (*p* = 0.032), whereas Ph.D. holders had significantly lower odds of perceiving the system as efficient (*p* = 0.024).

**Conclusion:**

This study reveals high satisfaction levels among middle-aged users of Wasfaty in Tabuk. In this convenience sample survey, participants’ satisfaction with the Wasfaty system was associated with perceptions of system efficiency, pharmacists’ counseling, and service availability. These findings should be interpreted with caution, as the study design does not allow for strong causal inferences or generalization to the wider population.

## Introduction

With Saudi Vision 2030 as a guiding framework, the Saudi national healthcare system has undergone dramatic transformation to address growing public demand, escalating healthcare costs, and a rising burden of chronic disease (Suleiman & Ming, 2025). Historically, the government sector delivered free healthcare services to all citizens, while the private sector later supplemented access—dependent on health insurance coverage or one’s ability to pay (Nair et al., 2024). A pivotal innovation aligned with Vision 2030 is the Wasfaty electronic prescription system, which enhances efficiency, precision, and convenience for both healthcare providers and patients (Alzahrani et al., 2024).

Wasfaty enables patients to fulfill prescriptions and obtain medical supplies through licensed community pharmacies. This initiative, one of several aimed at elevating healthcare standards, also offers short message service updates on prescription status, directing patients to the nearest pharmacy for free medication collection, re-dispensing, and even home delivery (AlGhadeer et al., 2023). By eliminating handwriting ambiguities and automatically checking for conflicting prescriptions, the system significantly reduces medication errors and enhances patient safety (Rasheed et al., 2024). Moreover, Wasfaty standardizes medication dispensing through digitized processes, ensuring access to high-quality pharmaceuticals and clear guidance on their use (Alkathiri et al., 2025).

Emulating other high-income nations, Saudi Arabia has shifted away from paper-based prescriptions toward a digital model (Alshammari et al., 2023). This transition has improved quality, safety, cost-effectiveness, and efficiency while reducing drug-related errors in both prescribing and dispensing phases (Alsahali et al., 2023). Community pharmacists have attributed most dispensing mistakes to illegible doctor’s handwriting, underscoring the importance of printed or electronic prescriptions in mitigating these risks (Alanazi et al., 2024). Wasfaty system directly addresses this challenge.

Patients’ demographics, behaviors, and prior experiences influence their utilization of health services (Vaz et al., 2023). A key indicator of healthcare uptake is user satisfaction, which directly affects future use of services (Ferreira et al., 2023). Dissatisfaction—especially due to issues with obtaining or using prescribed medication—can deteriorate health outcomes and, paradoxically, increase healthcare costs, negating the efficiencies introduced by new systems (Alzahrani et al., 2024). Thus, evaluating patient satisfaction, particularly with innovations like Wasfaty, is vital.

Poorly designed e-prescription systems can harm healthcare quality and customer satisfaction, ultimately undermining implementation (Hareem et al., 2023). While a recent study in Tabuk demonstrated high overall satisfaction with Wasfaty, its survey primarily included younger adults (18–30), who tend to be more digitally adept (Prabahar et al., 2025). This highlights a knowledge gap concerning middle-aged adults (40–59), a demographic that often bears a higher prevalence of chronic disease and frequent Wasfaty use. Accordingly, this study aims to assess the satisfaction and perspectives of middle-aged users regarding the Wasfaty system in Tabuk. The secondary aim was to examine the association between satisfaction and demographic characteristics such as age, gender, education, occupation, and presence of chronic diseases.

## Materials & Methods

### Study design

This cross-sectional study employed convenience sampling.

### Population and setting

Participants aged 40–59 years who were using the Wasfaty system were recruited from selected community pharmacies in Tabuk, Saudi Arabia.

### Sample size

The sample size was calculated using Sample Size Calculator (https://www.calculator.net/sample-size-calculator.html) (calculator.net, 2024).

Based on a previous study in Tabuk that reported approximately 80% satisfaction among Wasfaty users (Prabahar et al., 2025), the expected prevalence of satisfaction with the Wasfaty system was set at 80%, with a 5% margin of error and a 95% confidence level. The initial estimated sample size was 246. After applying the finite population correction for Tabuk’s population of 710,000 (GMI Research Team, 2025), the adjusted sample size remained approximately 246. To improve precision and account for potential non-response, the final target sample was increased to 384 participants, and 400 completed responses were included in the analysis.

### Eligibility criteria

Inclusion: Middle-aged adults (40–59 years) residing in Tabuk, Saudi Arabia, who actively use the Wasfaty system and consent to participate.

Exclusion: Non-Saudi nationals and individuals unwilling to provide informed consent.

### Ethical approval

The study protocol received approval from University of Tabuk Local Research Ethics Committee (Approval No. UT-324-162-2023) and adhered to the principles of the Declaration of Helsinki.

### Selection of community pharmacies

A convenience sampling technique was employed to recruit participants from 25 community pharmacies registered under the Wasfaty program and located in urban areas of Tabuk. Pharmacies were selected based on their participation in the Wasfaty network and their willingness to allow data collection during the study period. Within each participating pharmacy, eligible middle-aged users of the Wasfaty system were approached.

### Study procedure

From February to April 2025, the trained researcher approached potential participants at selected community pharmacies across urban areas of Tabuk, during weekdays (Sunday to Thursday between 9 am and 2 pm) and briefly explained the study objectives. Individuals who met the inclusion criteria (middle-aged adults, 40–59 years) were invited to participate. Participants who provided verbal consent were either given a paper questionnaire to complete on-site or provided a secure online link to complete the survey at home, depending on their preference. Instructions for completing the questionnaire were clearly provided, emphasizing confidentiality and voluntary participation. No reminders were sent for online completion, and participants completed the survey independently. Convenience sampling was used, meaning participants were recruited based on their availability and willingness to participate at the time of visit.

Participants anonymously completed the Arabic-language questionnaire, previously validated in an earlier Tabuk study, covering their perceptions and satisfaction with the Wasfaty system (Prabahar et al., 2025). Its content validity was established through expert review by a panel of five pharmacists and two academic researchers specializing in pharmaceutical care and health informatics. Reliability testing during the pilot phase yielded a Cronbach’s alpha of 0.84, indicating good internal consistency. For the present study, minor wording modifications were made to improve clarity and relevance for middle-aged adults (aged 40–59), and the instrument was re-assessed for face validity by two bilingual experts to ensure linguistic and cultural appropriateness of the Arabic version.

The instrument comprised two sections:

(1) Demographics: gender, age, occupation, education level, and chronic disease status.

(2) System feedback: eight yes/no questions evaluating Wasfaty-related services and one yes/no item assessing overall satisfaction.

### Handling of missing data

During data collection, measures were taken to minimize missing responses. For paper-based questionnaires, the researcher reviewed completed forms on-site and politely asked participants to fill in any unanswered items before submission. For the online version, all questions were set as mandatory fields (indicated by a red asterisk), preventing submission of incomplete responses. Consequently, the dataset contained no missing values for the analyzed variables.

### Statistical analysis

Data were processed using SPSS v27 (IBM Corp., Armonk, NY, USA). Descriptive statistics (frequencies and percentages) described participant characteristics and response frequencies. Logistic regression analysis calculated crude odds ratios (CORs) and adjusted odds ratios (AORs) with 95% confidence intervals (CI). In the multivariate logistic regression models, variables that showed significant associations in the bivariate analysis (*p* < 0.10) were entered simultaneously to control for potential confounding. The adjusted models included key demographic covariates, age group, gender, education level, employment status, and chronic disease status, as potential confounders, along with all perception variables conceptually related to satisfaction (*e.g.*, pharmacist counseling, service availability, and system efficiency). AORs with 95% CIs were computed to identify independent predictors of satisfaction with the Wasfaty system. Statistical significance was set at *p* < 0.05. Given the exploratory nature of this study, no formal correction for multiple comparisons (*e.g.*, Bonferroni adjustment) was applied. However, interpretation of *p*-values was made cautiously, focusing on the strength and consistency of observed associations rather than on isolated statistical significance.

## Results

Of the 412 eligible individuals approached, 400 consented and completed the questionnaire, resulting in a response rate of 97%.

### Participants’ demographic details

Table 1 outlines the demographic characteristics of the 400 study participants recruited from community pharmacies in Tabuk. This summary encompasses occupation, age, gender, education level, and chronic disease status, offering a comprehensive profile of the target population. Most participants were male (54.3%, *n* = 217). As all participants were middle-aged adults, the majority (61%, *n* = 244) fell within the 40–49 age range.

**Table 1:** Demographic characteristics of the participants.

**Demographic characteristics**	**Categories**	***n* = 400**	**%**
Gender	Male	217	54.3
	Female	183	45.8
Age	40–49	244	61
	50–59	156	39
Education	Primary	25	6.3
	Secondary	25	6.3
	Diploma	63	15.8
	Bachelor’s	234	58.5
	Master’s	42	10.5
	Ph.D	11	2.8
Occupation	Employed	318	79.5
	Unemployed	82	20.5
Chronic disease	Yes	253	63.3
	No	147	36.8

### Satisfaction with the wasfaty system

**Figure 1:**
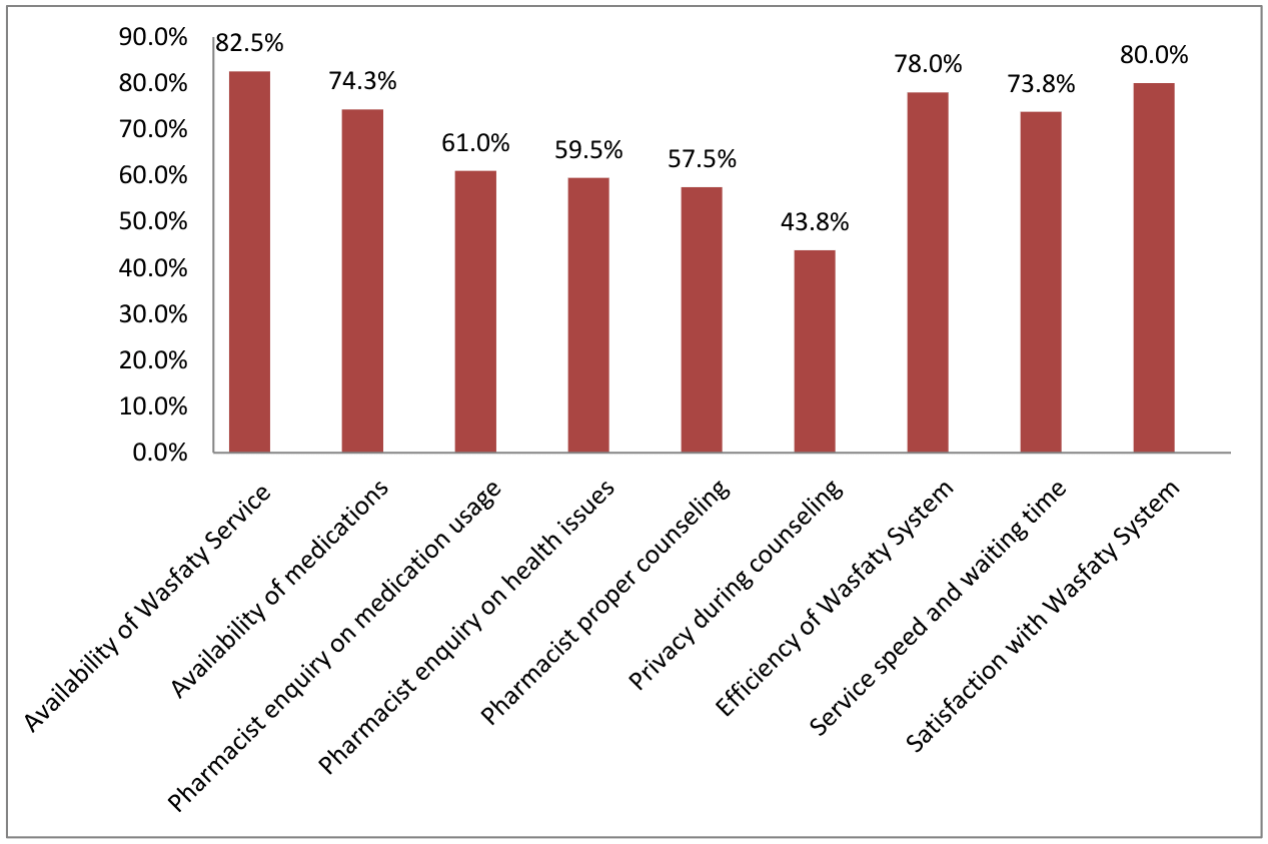
Participants’ perspectives and satisfaction with the Wasfaty System.

Figure 1 presents participants’ viewpoints and satisfaction levels regarding the Wasfaty system. Overall, 80% (*n* = 320) of respondents reported satisfaction with its services. Additionally, 82.5% (*n* = 330) confirmed that their nearest pharmacy participated in the Wasfaty program.

### Odds ratios for perspective variables and satisfaction with the Wasfaty system

Table 2 presents COR, AOR, *p*-values, and 95% CI for various factors influencing satisfaction with the Wasfaty system. In the unadjusted (crude) analysis, every variable demonstrated a significant positive association with satisfaction (*p* < 0.05), indicating that higher odds of satisfaction were seen across the board.

**Table 2:** Odds ratios for perspective variables and satisfaction with the Wasfaty system.

**Variables**	**COR [95% CI]**	**p-value**	**AOR [95% CI]**	***p*-value**
Availability of Wasfaty Service near your location	5.882 [2.708–12.773]	**<0.001**	3.254 [1.034–10.237]	**0.044**
Availability of medications	6.133 [2.963–12.696]	**<0.001**	2.315 [0.778–6.882]	0.131
Pharmacists’ enquiry on medication usage	7.552 [3.450–16.529]	**<0.001**	1.684 [0.502–5.653]	0.399
Pharmacists’ enquiry on health issues	3.895 [1.902–7.975]	**<0.001**	1.189 [0.387–3.652]	0.763
Pharmacists’ counseling about proper use of medications	8.732 [3.789–20.123]	**<0.001**	5.741 [1.128–29.227]	**0.035**
Privacy during consultation	10.450 [3.571–30.583]	**<0.001**	0.802 [0.104–6.173]	0.832
Efficiency of Wasfaty system	43.718 [17.263–74.721]	**<0.001**	20.597 [6.279–67.566]	**<0.001**
Service speed and waiting time	5.892 [2.854–12.165]	**<0.001**	0.554 [0.164–1.869]	0.341

**Notes.**

*p* < 0.05 - Significant-indicated in bold.

After adjusting for demographic covariates (age, gender, education, employment, and chronic disease status) and other perception variables in the multivariate model, three factors remained significantly linked with increased satisfaction:

System efficiency: Participants who perceived the Wasfaty system as efficient were far more likely to report satisfaction (AOR =20.597, *p* < 0.001).

Pharmacist counseling: Those who received advice from pharmacists regarding appropriate medication use showed a significant uptick in satisfaction.

Local service accessibility: Having access to the Wasfaty service at the nearest pharmacy was also a key predictor of satisfaction.

### Odds ratios for demographic variables and satisfaction with the Wasfaty system

Table 3 displays the COR, AOR, alongside 95% CI and *p*-values, evaluating the association between demographic characteristics and satisfaction with the Wasfaty system. Among middle-aged adults, none of the demographic factors showed a statistically significant influence on their satisfaction levels with Wasfaty.

**Table 3:** Odds ratios for demographic variables and satisfaction with the Wasfaty system.

**Demographic characteristics**	**Categories**	**COR [95% CI]**	***p*-value**	**AOR [95% CI]**	***p*-value**
Gender	Male	0.909 [0.462–1.789]	0.782	0.976 [0.467–2.039]	0.948
	Female				
Age	40–49	1.219 [0.614–2.420]	0.572	1.156 [0.548–2.437]	0.704
	50–59				
Education	Primary				
	Secondary	1.457 [0.369–5.752]	0.591	1.513 [0.364–6.295]	0.569
	Diploma	0.938 [0.206–4.270]	0.934	0.974 [0.202–4.692]	0.974
	Bachelor’s	1.350 [0.250–7.278]	0.727	1.359 [0.243–7.615]	0.727
	Master’s	1.500 [0.123–18.363]	0.751	1.530 [0.121–19.392]	0.743
	Ph.D	0.480 [0.087–2.645]	0.399	0.576 [0.086-3.850]	0.569
Occupation	Employed	1.508 [0.686–3.317]	0.307	1.201 [0.497–2.904]	0.684
	Unemployed				
Chronic disease	Yes	0.926 [0.461–1.861]	0.830	1.047 [0.499–2.1943]	0.904
	No				

### Odds ratios for demographic variables and statistically significant perspective variables with the satisfaction of Wasfaty system

Table 4 displays the OR evaluating the association between demographic characteristics and statistically significant perspective variables with the satisfaction of Wasfaty system. As observed from the *p*-value, male gender had statistically significant higher odds perspective with regard to pharmacists’ counseling about proper use of medications (*p* = 0.032), and Ph.D graduates had statistically significant lower odds perspective with regard to efficiency of Wasfaty system (*p* = 0.024).

**Table 4:** Odds ratios for demographic variables and statistically significant perspective variables with the satisfaction of Wasfaty system.

**Demographic characteristics**	**Categories**	**Availability of Wasfaty service near your location**		**Pharmacists’ counseling about proper use of medications**		**Efficiency of Wasfaty system**	
		**OR [95% CI]**	***p*-value**	**OR [95% CI]**	***p*-value**	**OR [95% CI]**	***p*-value**
Gender	Male	0.760 [0.373–1.547]	0.449	1.843 [1.054–3.223]	**0.032**	0.717 [0.372–1.382]	0.321
	Female						
Age	40-49	1.613 [0.790–3.297]	0.190	1.107 [0.632–1.939]	0.721	1.418 [0.732–2.747]	0.300
	50-59						
Education	Primary						
	Secondary	1.545 [0.390–6.118]	0.536	1.762 [0.559–5.557]	0.334	1.304 [0.332–5.120]	0.703
	Diploma	1.350 [0.282–6.452]	0.707	1.583 [0.436–5.755]	0.485	1.114 [0.240–5.180]	0.890
	Bachelor’s	3.000 [0.430–20.951]	0.268	1.400 [0.354–5.542]	0.632	1.350 [0.250–7.278]	0.727
	Master’s	0.676 [0.054–6.160]	0.754	2.333 [0.310–17.545]	0.410	0.980 [0.373-12.547]	0.921
	Ph.D	0.350 [0.065–1.895]	0.223	0.857 [0.214–4.674]	0.782	0.133 [0.023–0.765]	**0.024**
Occupation	Employed	1.571 [0.693–3.563]	0.279	0.673 [0.335–1.352]	0.266	1.514 [0.704–3.256]	0.288
	Unemployed						
Chronic disease	Yes	1.084 [0.516–2.277]	0.831	1.055 [0.597–1.862]	0.854	0.778 [0.398–1.517]	0.461
	No						

**Notes.**

*p* < 0.05 - Significant-indicated in bold.

## Discussion

To enhance efficiency and lower healthcare delivery costs, Saudi Arabia has transitioned pharmaceutical services away from government-operated primary health care centers to private community pharmacies through the adoption of an electronic prescribing platform known as Wasfaty. To the best of available knowledge, this study is the first to explore satisfaction with the Wasfaty system among a convenience sample of middle-aged adults attending community pharmacies in Tabuk. In this regional sample, 80% of middle-aged adults reported satisfaction with the Wasfaty system. This satisfaction level is slightly higher than that reported in a recently conducted study in Tabuk (Prabahar et al., 2025). About 63.3% of the middle-aged adults in this study had chronic diseases, and the regression analysis revealed that pharmacists’ counseling about proper use of medications was one of the perspective variables showing higher odds, which positively correlated with the satisfaction level. This may be the reason for the middle-aged adults exhibiting higher satisfaction with the Wasfaty system. A cross-sectional study in Punjab also found that the majority of patients were satisfied with the pharmacist’s explanation of the purpose of their prescribed medications (Alanazi et al., 2023).

With regard to the satisfaction with the Wasfaty system, a strong majority (80%) reported satisfaction, with 82.5% confirming that their nearest pharmacy participated in Wasfaty. A positive perspective was observed with the efficiency of Wasfaty system (78%), availability of medications in the pharmacy (74.3%), and service speed and waiting time (73.8%). This aligns with broader patient surveys in Saudi Arabia, which report high satisfaction across pharmacy access, counseling quality, waiting times, and privacy (Almaghaslah et al., 2022).

In the multivariate analysis, three factors remained significant predictors of satisfaction: Efficient system performance, Pharmacist counseling on proper medication use, and Local service accessibility. These strongly echo patient-reported priorities in other studies: pharmacists’ expertise, service accessibility, and system reliability are pivotal (Rasheed et al., 2024). The system’s efficiency factor is especially critical, matching pharmacists’ concerns about usability and workload. Slow/unintuitive systems can diminish satisfaction from both user and provider perspectives (Clarke et al., 2020). Pharmacist counseling remains central; patients consistently emphasize the value of clear communication on medication usage (Rusu et al., 2022). Saudi pharmacists demonstrated a strong enthusiasm for continuing education and to update their knowledge, convinced that it considerably enhances their professional performance (Alharthi et al., 2021). Access to nearby pharmacies carrying Wasfaty prescriptions ensures convenience and continuity—this aligns with high satisfaction scores for pharmacy accessibility (Alkathiri et al., 2025). A Qatar-based study also found that patient satisfaction is positively influenced by the speed of service, pharmacists’ attitudes, quality of medication counseling, as well as the pharmacy’s location and the comfort of its waiting area (Khudair & Raza, 2013). A very high odds of satisfaction observed with the efficiency of Wasfaty system indicate that the middle-aged adults were satisfied with the clarity of Wasfaty’s instructions, and ease of use of the application. It results in faster seamless inter-provider communication to reduce frustration (Leemanza & Kristin, 2024).

In this Tabuk-based sample of middle-aged adults, education, gender, age, employment, and chronic disease status showed no effect on the satisfaction level. This is consistent with other research that typically finds demographic traits have limited predictive power compared to service experience factors (Alzahrani et al., 2024). However, the regression analysis of demographic variables and statistically significant perspective variables with the satisfaction of Wasfaty system revealed that male gender had statistically significant higher odds perspective with regard to pharmacists’ counseling about proper use of medications, and Ph.D graduates had statistically significant lower odds perspective with regard to efficiency of Wasfaty system. Men were more likely than women to adhere to their chronic medications, which may partly explain why men demonstrate higher odds of positively viewing pharmacists’ counseling (Manteuffel et al., 2014). There may be two potential reasons for the Ph.D graduates exhibiting lower odds perspective with regard to efficiency of Wasfaty system. One reason is that the sample of Ph.D graduates within this convenience sample was very small (2.8%), which could have affected the validity of the results. Another reason might be that highly educated individuals tend to expect more advanced features to be included in the Wasfaty system. Patients with higher education levels sought more in-depth information about their medications and were more inclined to scrutinize various aspects of pharmacy services that might have led to lower odds perspective (Ng et al., 2021). In an Ethiopian study, both educational level and occupation were statistically significantly associated with clients’ level of perception, and satisfaction was also significantly linked to educational level (Teshome Kefale, Hagos Atsebah & Ayele Mega, 2016). Various factors might have influenced the study results including cultural and demographic factors, customers’ expectation, and quality of service provided.

### Limitations

As this study was conducted exclusively in Tabuk, the findings reflect regional user experiences rather than national-level perspectives. This study used convenience sampling from a limited number of community pharmacies registered in the Wasfaty network within Tabuk, which may introduce selection bias. Pharmacies that agreed to participate might have more engaged or satisfied customers, potentially inflating satisfaction levels. Therefore, the findings may not be generalizable to all middle-aged adults using the Wasfaty system across other regions of Saudi Arabia. Future studies using random sampling and multi-center designs are recommended to enhance representativeness.

Although the response rate was high (97%), non-respondents and those who declined participation may differ in their perceptions, introducing potential volunteer bias. In addition, as data were self-reported, it was not possible to independently validate the accuracy or truthfulness of participants’ responses, which could lead to information bias.

Response bias may also have influenced the results, as participants who agreed to complete the questionnaire might have been more engaged or generally more satisfied with the Wasfaty services than non-respondents. Social desirability bias could also have contributed to higher reported satisfaction levels, since participants might have felt reluctant to express negative opinions about a national healthcare program. This potential overestimation of satisfaction should be considered when interpreting the findings. To minimize such effects, the survey was administered anonymously, and participants were assured that their responses would not affect their access to services; nonetheless, some degree of response bias cannot be entirely excluded. Multiple comparisons were conducted across several demographic and perception variables, which may increase the likelihood of Type I error. Although no formal *p*-value adjustment was applied, findings were interpreted cautiously as indicative rather than confirmatory.

### Areas of strength and recommendations

• Support pharmacists in developing strong counseling skills through ongoing training and adequate consultation time.
• Expand the number and geographic distribution of affiliated pharmacies within similar urban settings to address accessibility concerns.
• Simplify system navigation, prescription error alerts, and pharmacy–physician messaging to enhance user experience in comparable Wasfaty implementation contexts.

## Conclusions

This study suggests that middle-aged adults recruited from selected community pharmacies in Tabuk, Saudi Arabia generally reported high satisfaction with the Wasfaty system. In this convenience-sampled, regional cohort, perceived system efficiency, pharmacist counseling, and service availability at local pharmacies were significantly associated with higher satisfaction levels. These findings indicate that, within similar regional and pharmacy-based contexts, operational aspects of the system may play a more influential role in shaping satisfaction than demographic characteristics.

However, as this was a cross-sectional survey based on self-reported data from a convenience sample, the findings should be interpreted cautiously and should not be generalized to all Wasfaty users across Saudi Arabia. Future studies employing random sampling, multi-region designs, and anonymized electronic data collection are needed to confirm and extend these findings.

## Supplemental Information

10.7717/peerj.21011/supp-1 Supplemental Information 1 Strobe checklist

10.7717/peerj.21011/supp-2 Supplemental Information 2 Raw data

10.7717/peerj.21011/supp-3 Supplemental Information 3 Data collection form - English

10.7717/peerj.21011/supp-4 Supplemental Information 4 Data collection form - Arabic

## Additional Information and Declarations

### Data Availability

The following information was supplied regarding data availability: The raw data are available in the Supplemental File.
