# Supplementary material for: Exploring middle-aged adults’ satisfaction with the Wasfaty electronic prescription system: a cross-sectional study in Tabuk, Saudi Arabia
Source: PeerJ. 2026 Mar 23;14:e21011. doi: 10.7717/peerj.21011 (PMC13020429; doi:10.7717/peerj.21011)
Supplement: Supplemental Information 1 [file peerj-14-21011-s001.pdf]

STROBE Statement—checklist of items that should be included in reports of observational studies

	Item No.	Recommendation	Page No.	Relevant text from manuscript
**Title and abstract**	1	(a) Indicate the study's design with a commonly used term in the title or the abstract	1	Cross-sectional survey exploring middle-aged adults' satisfaction with Wasfaty
		(*b*) Provide in the abstract an informative and balanced summary of what was done and what was found	1	A cross-sectional survey was conducted among middle-aged users of the Wasfaty System. This study reveals high satisfaction levels among middle-aged users of Wasfaty. The main factors driving satisfaction are system efficiency, pharmacists' counseling, and local service availability.
**Introduction**				
Background/rationale	2	Explain the scientific background and rationale for the investigation being reported	2	Poorly designed e-prescription systems can harm healthcare quality and customer satisfaction, ultimately undermining implementation. While a recent study in Tabuk demonstrated high overall satisfaction with Wasfaty, its survey primarily included younger adults (18–30), who tend to be more digitally adept. This highlights a knowledge gap

				concerning middle-aged adults (40–59)—a demographic that often bears a higher prevalence of chronic disease and frequent Wasfaty use.
Objectives	3	State specific objectives, including any prespecified hypotheses	2	Our study aims to assess the satisfaction and perspectives of middle-aged users regarding the Wasfaty system.
**Methods**				
Study design	4	Present key elements of study design early in the paper	3	This cross-sectional study employed convenience sampling.
Setting	5	Describe the setting, locations, and relevant dates, including periods of recruitment, exposure, follow-up, and data collection	3	Patients aged 40–59 using the Wasfaty System were recruited from the community pharmacies to complete the self-administered Arabic questionnaire.
Participants	6	(*a*) *Cohort study*—Give the eligibility criteria, and the sources and methods of selection of participants. Describe methods of follow-up<br>*Case-control study*—Give the eligibility criteria, and the sources and methods of case ascertainment and control selection. Give the rationale for the choice of cases and controls<br>*Cross-sectional study*—Give the eligibility criteria, and the sources and methods of selection of participants	3	Inclusion: Middle-aged adults (40–59 years) residing in Tabuk, Saudi Arabia, who actively use the Wasfaty system and consent to participate.<br>Exclusion: Non-Saudi nationals and individuals unwilling to provide informed consent.
		(*b*) *Cohort study*—For matched studies, give matching criteria and number of exposed and unexposed<br>*Case-control study*—For matched studies, give matching criteria and the number of controls per case		

Variables	7	Clearly define all outcomes, exposures, predictors, potential confounders, and effect modifiers. Give diagnostic criteria, if applicable	3	From February to April 2025, individuals visiting selected community pharmacies were informed about the study and invited to participate. Upon agreement, they provided written informed consent. Confidentiality of responses was assured. Participants anonymously completed the Arabic-language questionnaire—previously validated in an earlier Tabuk study—covering their perceptions and satisfaction with the Wasfaty System (Prabahar et al., 2025). The instrument comprised two sections:<br>(1) Demographics: gender, age, occupation, education level, and chronic disease status.<br>(2) System feedback: eight yes/no questions evaluating Wasfaty-related services and one yes/no item assessing overall satisfaction.
Data sources/ measurement	8*	For each variable of interest, give sources of data and details of methods of assessment (measurement). Describe comparability of assessment methods if there is more than one group	3	Participants anonymously completed the Arabic-language questionnaire—previously validated in an earlier Tabuk study—covering their

				perceptions and satisfaction with the Wasfaty System (Prabahar et al., 2025). The instrument comprised two sections:<br>(1) Demographics: gender, age, occupation, education level, and chronic disease status.<br>(2) System feedback: eight yes/no questions evaluating Wasfaty-related services and one yes/no item assessing overall satisfaction.
Bias	9	Describe any efforts to address potential sources of bias	3	Only Saudi Nationals who use Wasfaty service were selected.
Study size	10	Explain how the study size was arrived at	3	The sample size was calculated using a computer formula that took into account Tabuk city's total Saudi population (710000). With a 95% confidence interval and a 5% margin of error (Sample Size Calculator), the required sample size was determined to be 384.

Continued on next page

Quantitative variables	11	Explain how quantitative variables were handled in the analyses. If applicable, describe which groupings were chosen and why	3	Descriptive statistics (frequencies and percentages) described participant characteristics and response frequencies.
Statistical methods	12	(*a*) Describe all statistical methods, including those used to control for confounding	3	Logistic regression analysis calculated crude odds ratios (CORs) and adjusted odds ratios (AORs) with 95% confidence intervals (CI). Statistical significance was set at $p < 0.05$.
		(*b*) Describe any methods used to examine subgroups and interactions		
		(*c*) Explain how missing data were addressed	3	The participants were directly contacted and if any missing data, they were asked to fill it up.
		(*d*) *Cohort study*—If applicable, explain how loss to follow-up was addressed<br>*Case-control study*—If applicable, explain how matching of cases and controls was addressed<br>*Cross-sectional study*—If applicable, describe analytical methods taking account of sampling strategy	3	Sample size calculator
		(*e*) Describe any sensitivity analyses		
**Results**				
Participants	13*	(a) Report numbers of individuals at each stage of study—eg numbers potentially eligible, examined for eligibility, confirmed eligible, included in the study, completing follow-up, and analysed	-	
		(b) Give reasons for non-participation at each stage	-	
		(c) Consider use of a flow diagram	-	
Descriptive data	14*	(a) Give characteristics of study participants (eg demographic, clinical, social) and information on exposures and potential confounders	3	Most participants were male (54.3%, n = 114). As all participants were middle-aged adults, the majority (61%, n = 128) fell within the 40–49 age range.
		(b) Indicate number of participants with missing data for each variable of interest	-	
		(c) *Cohort study*—Summarise follow-up time (eg, average and total amount)		

Outcome data	15*	*Cohort study*—Report numbers of outcome events or summary measures over time		
		Case-control study—Report numbers in each exposure category, or summary measures of exposure		
		Cross-sectional study—Report numbers of outcome events or summary measures	4	Overall, 80% of respondents reported satisfaction with its services. Additionally, the majority (82.4%) confirmed that their nearest pharmacy participated in the Wasfaty program.
Main results	16	(*a*) Give unadjusted estimates and, if applicable, confounder-adjusted estimates and their precision (eg, 95% confidence interval). Make clear which confounders were adjusted for and why they were included	4	In the unadjusted (crude) analysis, every variable demonstrated a significant positive association with satisfaction ($p < 0.05$), indicating that higher odds of satisfaction were seen across the board.<br>After adjusting for confounders, three factors remained significantly linked with increased satisfaction:<br>System Efficiency: Participants who perceived the Wasfaty System as efficient were far more likely to report satisfaction (AOR = 20.597, $p < 0.001$).<br>Pharmacist Counseling: Those who received advice from pharmacists regarding appropriate medication use showed a significant uptick in satisfaction.<br>Local Service Accessibility: Having access to the Wasfaty service at the nearest pharmacy was also a key predictor of satisfaction.

	(*b*) Report category boundaries when continuous variables were categorized	4	The odds ratio evaluating the association between demographic characteristics and statistically significant perspective variables with the satisfaction of Wasfaty System were displayed. As observed from the p-value, male gender had statistically significant higher odds perspective with regard to pharmacists' counseling about proper use of medications (p=0.032), and Ph.D graduates had statistically significant lower odds perspective with regard to efficiency of Wasfaty System (p=0.024).
	(*c*) If relevant, consider translating estimates of relative risk into absolute risk for a meaningful time period	-	

Continued on next page

Other analyses	17	Report other analyses done—eg analyses of subgroups and interactions, and sensitivity analyses	-	
**Discussion**				
Key results	18	Summarise key results with reference to study objectives	6	Our study confirms that the middle-aged adults demonstrated high satisfaction with the Wasfaty System, and system efficiency, pharmacist counseling, and service availability at local pharmacies are the primary drivers of patient satisfaction with Wasfaty. The positive significant association of perspective variables with the patient's satisfaction helps in promoting the quality of care provided to the patients. Demographic traits play a minimal role once these operational elements are optimized.
Limitations	19	Discuss limitations of the study, taking into account sources of potential bias or imprecision. Discuss both direction and magnitude of any potential bias	6	This study reflects the perspectives of consenting patients chosen from a limited set of pharmacies, which may introduce sampling bias. Additionally, respondents' answers could be influenced by response bias, potentially skewing the findings.
Interpretation	20	Give a cautious overall interpretation of results considering objectives, limitations, multiplicity of analyses, results from similar studies, and other relevant evidence	4	Our study findings revealed that 80% of middle-aged adults reported satisfaction with the Wasfaty System. The satisfaction level is slightly higher than that of the

				recently conducted study in Tabuk (Prabahar et al., 2025). About 63% of the middle-aged adults in our study had chronic diseases and the regression analysis revealed that pharmacists' counseling about proper use of medications was one of the perspective variable which showed higher odds (OR: 5.741) which positively correlated with the satisfaction level. This may be the reason for the middle-aged adults exhibiting higher satisfaction with the Wasfaty System. A cross-sectional study in Punjab also found that the majority of patients were satisfied with the pharmacist's explanation of the purpose of their prescribed medications (Alanazi et al., 2023).
Generalisability	21	Discuss the generalisability (external validity) of the study results	6	• Support pharmacists in developing strong counseling skills with ongoing training and adequate consultation time.<br>• Expand the number and geographic distribution of affiliated pharmacies to meet the critical "nearby pharmacy" need.<br>• Simplify system

				navigation, prescription error alerts, and built-in pharmacy–doctor messaging to enhance provider experience.
**Other information**				
Funding	22	Give the source of funding and the role of the funders for the present study and, if applicable, for the original study on which the present article is based	-	

*Give information separately for cases and controls in case-control studies and, if applicable, for exposed and unexposed groups in cohort and cross-sectional studies.

Note: An Explanation and Elaboration article discusses each checklist item and gives methodological background and published examples of transparent reporting. The STROBE checklist is best used in conjunction with this article (freely available on the Web sites of PLoS Medicine at http://www.plosmedicine.org/, Annals of Internal Medicine at http://www.annals.org/, and Epidemiology at http://www.epidem.com/). Information on the STROBE Initiative is available at www.strobe-statement.org.
