# Supplementary material for: Exploring middle-aged adults’ satisfaction with the Wasfaty electronic prescription system: a cross-sectional study in Tabuk, Saudi Arabia
Source: PeerJ. 2026 Mar 23;14:e21011. doi: 10.7717/peerj.21011 (PMC13020429; doi:10.7717/peerj.21011)
Supplement: Supplemental Information 3 [file peerj-14-21011-s003.pdf]

A questionnaire study on the extent of patient satisfaction with the application of my prescription in community pharmacies

Gender

- Male
- Female

Age

- 40-49
- 50-59

Educational level

- Have only completed primary school
- Have only completed high school
- Diploma
- Bachelor's degree
- Master's degree
- Doctorate degree

Occupation

- Unemployed
- Employed

Chronic diseases

- Yes
- No

Marital status

- Single
- Married
- Divorced
- Widow

S.No.	Patient satisfaction with the application of my prescription in community pharmacies	Yes	No
1.	Is the Wasfaty service available in a location close to where you live?		
2.	Did you find all your prescription medications available at the pharmacy?		
3.	Does the pharmacist enquire if you use any other		

	medicines?		
4.	Does the pharmacist enquire if you have any other health problems?		
5.	Did you feel that your pharmacist gave you detailed instructions about the medicines that were made available to you?		
6.	Was there privacy during your conversation with the pharmacist about your health condition?		
7.	Did Wasfaty app meet your needs faster than you expected?		
8.	Was Wasfaty able to save waiting time and provide a quick service?		
9.	Is your experience positive with the Wasfaty app?		
