# Supplementary material for: Exploring middle-aged adults’ satisfaction with the Wasfaty electronic prescription system: a cross-sectional study in Tabuk, Saudi Arabia
Source: PeerJ. 2026 Mar 23;14:e21011. doi: 10.7717/peerj.21011 (PMC13020429; doi:10.7717/peerj.21011)
Supplement: Supplemental Information 4 [file peerj-14-21011-s004.pdf]

استبانة دراسة عن مدى رضا المرضى عن تطبيق وصفتي في صيدليات المجتمع .

الجنس :

- أنثى
- ذكر

العمر:

- 40–49
- 50-59

المستوى التعليمي:

- حصلت على المدرسة الابتدائية فقط
- حصلت على المدرسة الثانوية فقط
- شهادة الدبلوم
- شهادة البكالوريوس
- شهادة الماجستير
- شهادة الدكتوراة

الوظيفة:

- غير موظف
- موظف

امراض مزمنة:

- نعم
- لا

الحالة الاجتماعية:

- اعزب
- متزوج
- مطلق
- ارملة

	رضا المرضى عن تطبيق وصفتي في صيدليات المجتمع	نعم	لا	
1	هل تتوفر خدمة وصفتي في موقع قريب من مكان اقامتك			
2	هل وجدت جميع الادوية الموصوفة لك متوفرة في الصيدلية			
3	هل يستفسر الصيدلي اذا كنت تستخدم أي ادوية أخرى			
4	هل يستفسر الصيدلي اذا كان لديك أي مشاكل صحية أخرى			
5	هل شعرت ان الصيدلي قدم لك إرشادات مفصلة حول الادوية الي تم توفيرها لك			
6	هل كان هناك خصوصية خلال محادثتك مع الصيدلي عن حالتك الصحية			
7	**هل ساهم تطبيق وصفتي في تلبية احتياجاتك بشكل أسرع مما كنت تتوقع؟**			
8	**هل استطاع تطبيق وصفتي حل مشكلة الانتظار وتقديم خدمة سريعة؟**			
9	**هل تعتبر تجربتك إيجابية مع تطبيق وصفتي ؟**			
